# In Situ Blue-Light-Induced Photocurable and Weavable Hydrogel Filament

**DOI:** 10.1021/acsomega.1c05354

**Published:** 2021-12-16

**Authors:** Chenglong Wang, Fan Meng, Luyang Qiao, Yuyan Xie, Xin Liu, Jinhuan Zheng

**Affiliations:** Engineering Research Center for Eco-Dyeing and Finishing of Textiles, Ministry of Education, Zhejiang Sci-Tech University, Hangzhou 310018, P. R. China

## Abstract

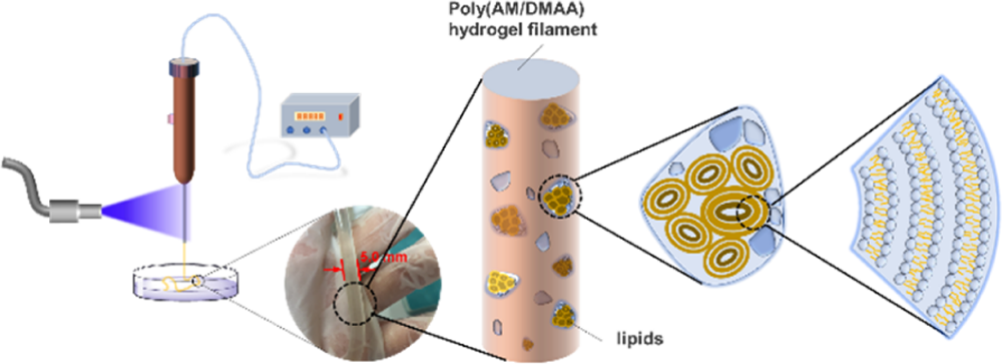

A self-lubricating hydrogel filament was achieved by establishing an in situ photocuring system and using camphorquinone/diphenyl iodonium hexafluorophosphate (CQ/DPI) as the blue-light photoinitiators, acrylamide (AM) and *N*,*N*-dimethylacrylamide (DMAA) as the monomers, polyethylene glycol diacrylate (PEGDA) as the cross-linker, and lecithin as the lipid lubricant. The blue-light photopolymerization efficiency and the photorheological properties of the hydrogel precursor were investigated by photodifferential scanning calorimetry and a photorheological system. With the increase of DMAA, the photopolymerization efficiency of the precursor improved, while the elasticity of poly(DMAA/AM) decreased accordingly. The physical cross-linking effect between lecithin and the poly(DMAA/AM) network led to improved polymerization properties and elasticity. The lipid-based boundary layer at the hydrogel surface endowed the self-lubrication of the hydrogel filament. The extruded hydrogel filaments exhibited excellent mechanical properties and weavability, which were expected to play a realistic role in soft robots and bioengineering.

## 1 Introduction

Hydrogel materials are popular in many fields, especially in soft robots and bioengineering scaffolds due to their three-dimensional network structure, excellent water absorbability, and water retention.^1−3^ The hydrogel filament is the fundamental architectural unit for many structures, for example, stacked 3D scaffolds and artificial blood vessels.

In 3D bioprinting, an extrusion-based object or scaffold can be deconstructed into several layers that are composed of one or more filaments. The structural stability of 3D-structured objects largely depends on the stacking mode and the physical and mechanical properties of the filaments. The fast manufacture of hydrogel filaments with excellent physical and mechanical properties is always a hotspot and difficult in relative fields. Heat-induced polymerization has been used to prepare poly(DMAA/sodium acrylate) hydrogel filaments with high water absorption and good mechanical properties, and it can absorb water over 1000 times heavier than itself.^4^ However, the synthesis time could be very long (normally more than 1 day).

A photo-cross-linkable hydrogel is considered an attractive material that is polymerized rapidly and enables process control in space and time.^5−7^ Photocuring technology is particularly suitable for constructing nanopatterned microstructures by using stereolithography or photomasking. Movable laser or high-resolution parallel light through the photomask was used as the exciting light source, and then, nano-microstructures were obtained by removing the uncured precursor.^8^ Such microstructures composed of nanohydrogel filaments were believed to be a powerful way for the conversion between complex and robust two-dimensional and three-dimensional structures involved in tissue engineering and optoelectronic applications. In order to obtain continuous hydrogel filaments, the photocuring system was always combined with an extrusion system.^9−11^ The location of the optical device and the fluid characteristics of the photocurable precursor become critical when a photo-cross-linkable system is applied to extrusion-based molding. Three types of photocuring processes, pre-cross-link (before extrusion), post-cross-link (after extrusion), or in situ-cross-link (during extrusion), were developed based on the different fluid characteristics of the photocurable precursor.^5,12,13^ Post-cross-link places high demands on the viscoelasticity of the photocurable precursor. The precursor with low viscosity and structure stability was first excluded in post-cross-link as it is very hard to maintain the original form after extrusion. Pre-cross-link and in situ-cross-link provide solution schemes for the precursor containing a large amount of low-molecular-weight monomers. The impact of low viscosity and structural stability is greatly inhibited when the hydrogel filament forms in a tube or a photopermeable capillary before extrusion. Although pre-cross-link and in situ-cross-linking could reduce the extrusion force, the precursor flowed prior to stabilization and might not maintain the filament structure. In addition, the low penetration of UV light and the adhesion between the hydrogel filament and the internal wall of the transparent capillary could also affect the extrusion efficiency.

In this work, blue light and its compatible efficient photoinitiators were selected as the photoinitiation system and then were devoted to develop a hydrogel with excellent physical and mechanical properties and high lubricity. A self-lubricating hydrogel filament was achieved by establishing an in situ photocuring system and using camphorquinone/diphenyl iodonium hexafluorophosphate (CQ/DPI) as the blue-light photoinitiators, acrylamide (AM) and *N*,*N*-dimethylacrylamide (DMAA) as the monomers, polyethylene glycol diacrylate (PEGDA) as the cross-linker, and lecithin as the lipid lubricant. The blue-light photopolymerization efficiency and photorheological properties of the hydrogel precursor were investigated by photo-differential scanning calorimetry (DSC) and a photorheological system. The basic mechanical properties of such hydrogel filaments were tested to ensure its weavability and application prospects.

## 2 Results and Discussion

### 2.1 Photopolymerization Kinetics

CQ, as a typical Norrish type II (hydrogen abstraction) photosensitizer, can form a stable triplet excited-state CQ* from the ground state after absorbing irradiation energy.^14−17^ In a CQ/DPI system, the cationic active center is produced by the photolysis of Ar–I in DPI by grabbing an electron from the surrounding molecules and then forming free radicals, including phenyl radicals and CQ^+•^. The combination of the two radicals plays a synergistic role and significantly improves the efficiency of radical photopolymerization.^18−20^

In the radical polymerization, many factors, for example, the monomer concentration and the gel effect, affect the photopolymerization efficiency together. The photopolymerization rates (*R*_p_, proportional to heat flow) of AM initiated by different dosages of CQ/DPI and double-bond conversion as a function of time are shown in Figure S1. *R*_p_ exhibits an upside-down “U-shaped” pattern, which involved initiation, propagation, and termination reactions. Degrees of double-bond conversion varied, again depending on *w*_CQ/DPI_, from 70 to nearly 80%, indicating an overall high photopolymerization efficiency.

**Figure 1:**
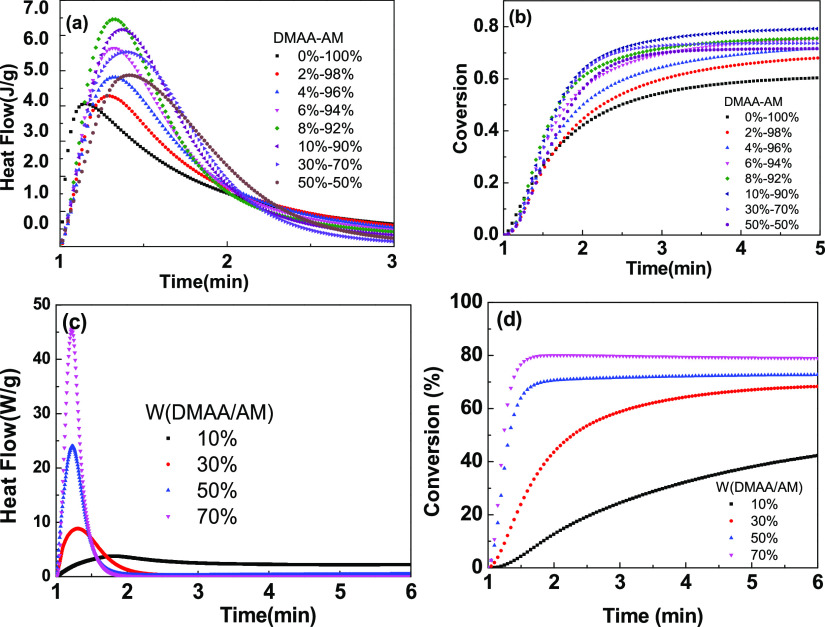
Heat flow during the polymerization and double-bond conversion of the polymerization precursor at different ratios of the monomers (a,b) and the total monomer amounts (c,d).

The blue-light polymerization performance of the hydrogel precursor with different mass ratios of DMAA/AM and monomer concentrations is shown in Figure 1. When AM acts alone, the hydrogel precursor displays a promising polymerization efficiency (approx. 60% of double-bond conversion). When DMAA was added into the AM system, the double-bond conversion significantly improved. When the proportion of DMAA continued to increase in the range of 0–8 wt %, the conversion showed a gradual increasing trend, which was mainly due to the coinitiation effect of the tertiary amino group on DMAA. At the same time, the time to reach the maximum polymerization rate was also delayed. According to the free volume effect, the volume shrinkage rate of the cross-linked network structure formed during the light curing process is much lower than the polymerization reaction rate, the volume shrinkage lags, and the components move freely in the excess free volume, increasing the collision probability and thus improving the double-bond conversion, as shown in Figure 1b. At the same time, when the proportion of DMAA was more than 10 wt %, the mole fraction of double bonds decreased significantly, and both *R*_P_ and the final double-bond conversion showed a downward trend. In addition, with the increase of the total monomer concentration, *R*_P_ increased, as shown in Figure 1c,d, which was consistent with the photopolymerization kinetics.

**Figure 2:**
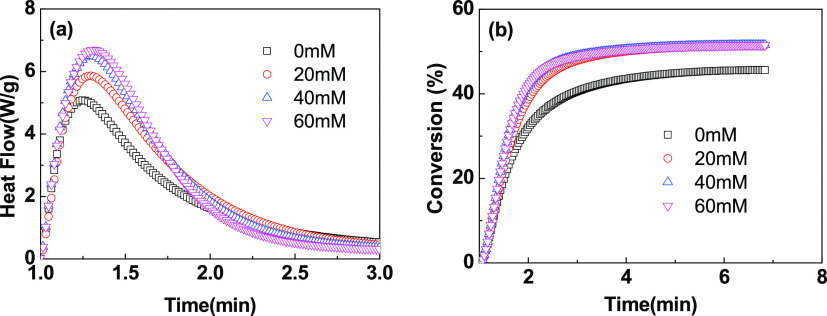
Heat flow during the polymerization (a) and double-bond conversion (b) of the polymerization precursor at different amounts of lecithin.

Further research studies on the effect of the addition of lecithin on photopolymerization performance showed that *R*_p_ and double-bond conversion improved with the increase of the concentration of lecithin, as shown in Figure 2. The synergism effect of lecithin on the photopolymerization efficiency could be attributed to the physical cross-linking between the polymer network and lecithin.

### 2.2 Photogelation Behavior

Gelation is an important characteristic in the process of photopolymerization and cross-linking of hydrogel precursors, which is very important for the formation of the internal structure of hydrogels.^21−23^ The critical reaction degree at which gelation occurs was regarded as the gelling point. At the gelling point, the physical properties of the system will undergo significant changes, such as autoaccelerating polymerization and increasing viscosity. The storage modulus (*G*′) is measured by the photorheological behavior, which is the energy stored by the material due to elastic deformation, while the loss modulus (*G*″) reflects the energy lost by the material in the form of heat due to viscous deformation.

**Figure 3:**
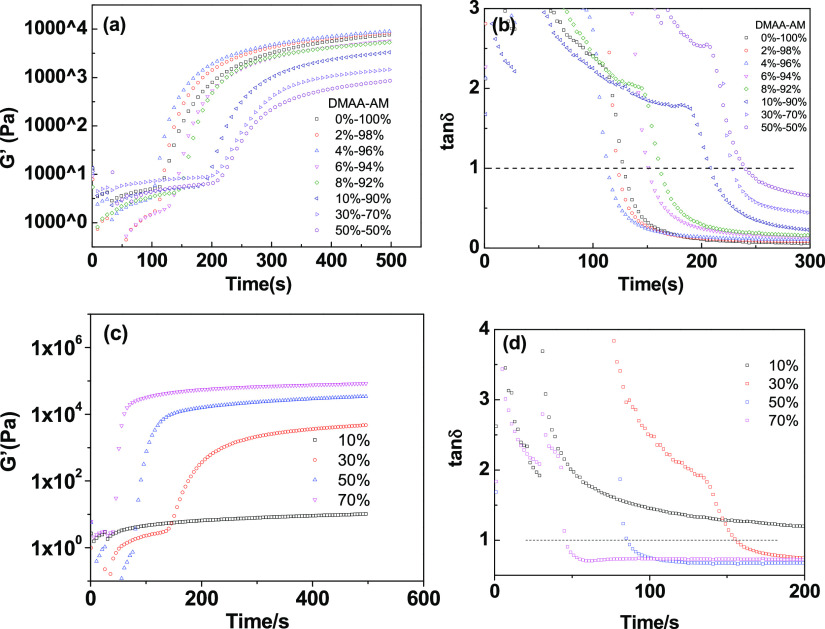
Storage modulus *G*′ and loss factor of the polymerization precursor at different ratios of the monomers (a,b) and total monomer amounts (c,d).

A series of important information, such as the modulus and the conversion time for gelling, through the real-time monitoring of the gel properties in the polymerization process by means of the photorheology system, are given in Figure 3. In the first 30 s without blue-light irradiation, the precursor fluid was mainly dominated by viscous characteristics (*G*″ ≥ *G*′). With further irradiation, a cross-linked network structure was formed between the monomers and cross-linking agents, and the hydrogel system started to exhibit elastic characteristics (*G*′ ≥ *G*″). When a small amount of DMAA was added, the storage modulus *G*′ of the precursor system in the presence of AM was improved, which could also be attributed to the coinitiation effect and cross-linking effect of DMAA; see Figure 3a. With the further increase of the dosages of DMAA, *G*′ decreased gradually due to the decreased cross-linking behavior caused by the decline of the mole fraction of the double bond. As shown in Figure 3b, the loss factor (tan δ) is defined as the ratio of *G*″ and *G*′, which decreased during the polymerization in general, indicating that the viscoelasticity of hydrogels changes dynamically.

Usually, we use an important parameter “mesh size ξ” in eq 1 to measure the network structure compactness of the hydrogels.^24−26^
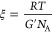
1where ξ is the mesh size, *R* is the gas constant, *T* is the temperature (k), Na is the Avogadro constant, and *G*′ is the modulus of elasticity (Pa).

It can be observed that as *G*′ increases, the smaller the ξ value, the higher the compactness of the spatial structure. As in Figure 1, the addition of DMAA also significantly improved the double-bond conversion and the spatial cross-linking degree of response. At the same time, with the increase of the DMAA ratio above 10 wt %, *G*′ decreased and the gel transition time (the time when tan δ equals to 1) gradually lengthened. In this process, the decrease of the molar ratio of the double bond led to a slower polymerization rate and slower formation of the cross-links. Meanwhile, the decrease of the conversion rate also reduced the compactness of the hydrogel microstructure, and thus, the storage modulus *G*′ decreased. As shown in Figure 3c,d, with the increase of the monomer concentration, the gel transition time shortened gradually, and the polymerization rate, the cross-linking density of the spatial structure, and *G*′ increased accordingly.

**Figure 4:**
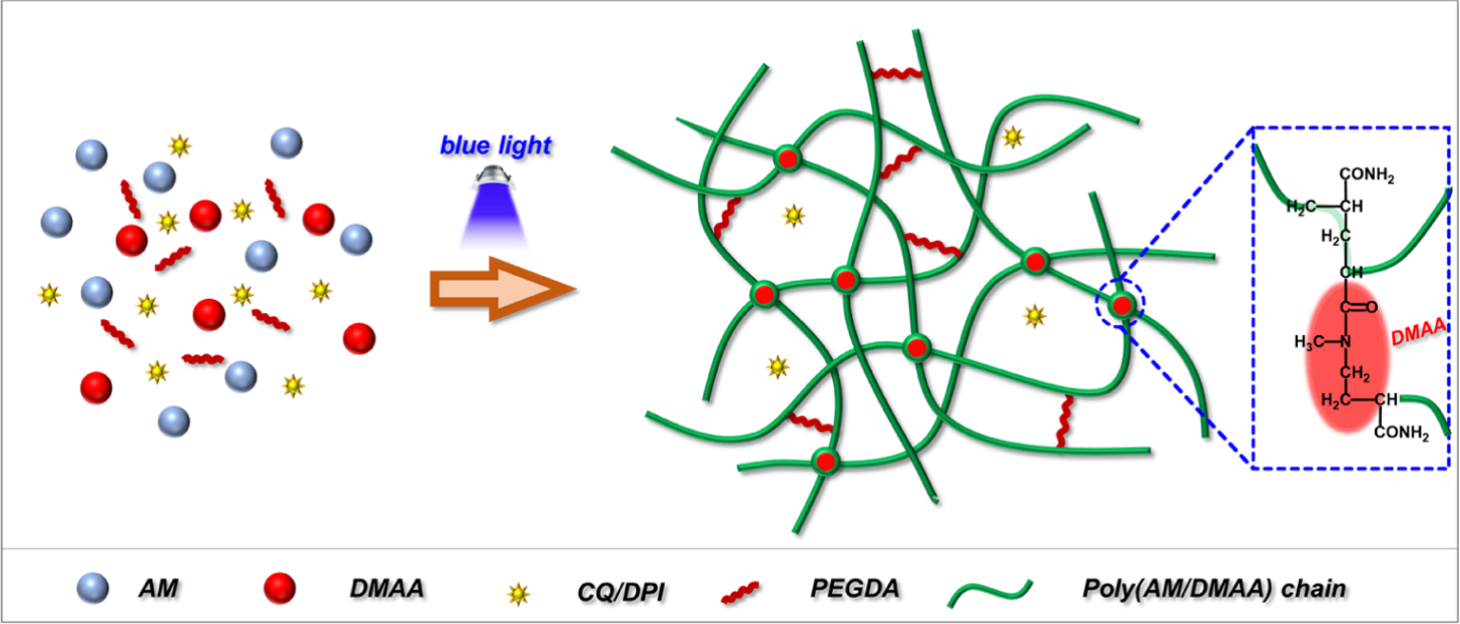
Cross-linking network structure of the poly(DMAA/AM) hydrogel.

The dual cross-linking mechanisms in the poly(DMAA/AM) structure are shown in Figure 4. Both PEGDA and DMAA acted as cross-linkers. Considering the subsequent spinning rate, polymerization rate, monomer utilization, and elastic deformation, 5% DMAA and 95% AM were chosen as the monomer composition.

**Figure 5:**
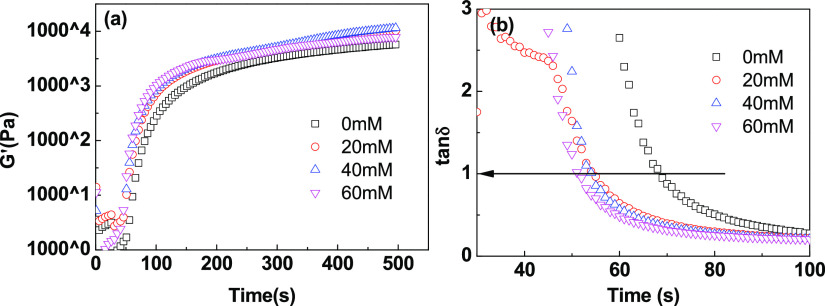
Storage modulus *G*′ (a) and loss factor (b) of the polymerization precursor at different amounts of lecithin.

In the meantime, a similar synergism effect of lecithin on the photopolymerization efficiency can also be found in Figure 5. It can be seen that *G*′ increased with the increase of the concentration of lecithin, while the gel transition time was shortened. The physical cross-linking between the polymer network and lecithin was also regarded as the cause of the increasing of gelation effect. The formation of physical cross-linking points promoted the gelation and viscoelastic transition of the poly(DMAA/SA) hydrogel.

### 2.3 Tensile Mechanical Properties of Poly(DMAA/SA) Hydrogels

**Figure 6:**
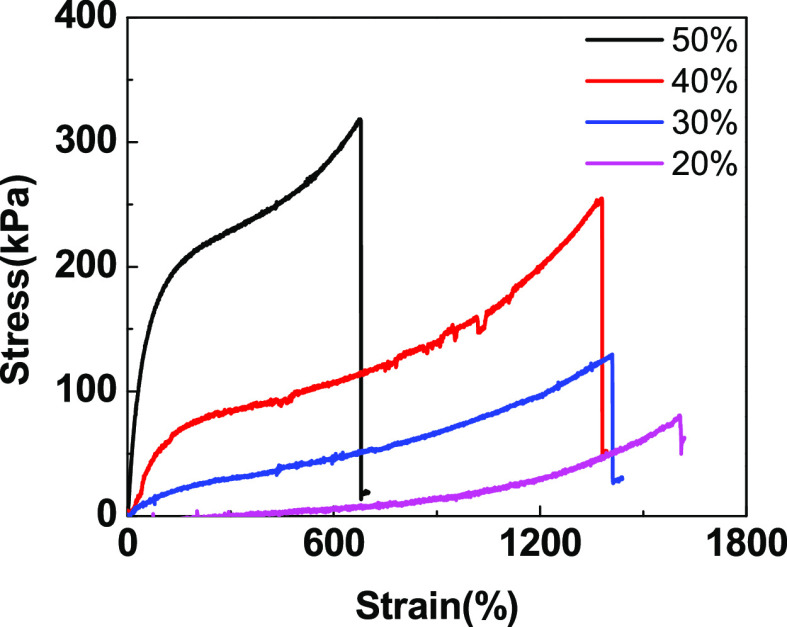
Stress–strain curve of the poly(DMAA/AM) hydrogel at different monomer amounts.

The tensile mechanical properties of the poly(DMAA/SA) hydrogels are shown in Figure 6. The large number of amide bonds in the poly(DMAA/SA) network structure provided the binding sites for the formation of hydrogen bond force. When poly(DMAA/SA) hydrogel was stretched, the intermolecular force between polymer networks was destroyed first, and new hydrogen bonds could be formed after the original hydrogen bonds were deconstructed. Therefore, the hydrogel presented good recovery performance within a certain range of stress. With the increase of the monomer concentration, the cross-linking density increased gradually, and greater stress was required to achieve the fracture structure.

### 2.4 Extrusion and Properties of Poly(DMAA/AM) Hydrogel Filaments

**Figure 7:**
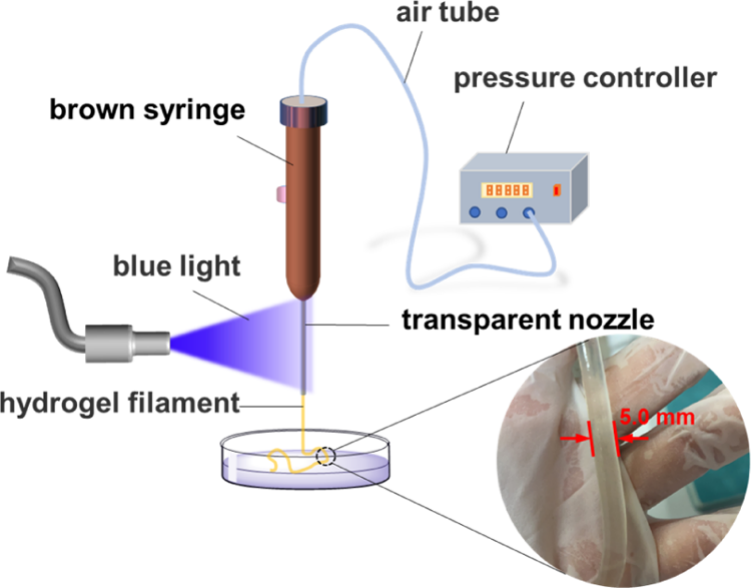
Extrusion devices and the extrusion mechanism of the poly(DMAA/AM) hydrogel filaments.

The possibility of in situ shaping was proved by the photocuring characteristics of the poly(DMAA/AM) hydrogel precursor. The extrusion devices for preparing poly(DMAA/AM) hydrogel filaments and their extrusion mechanism are shown in Figure 7. By controlling a series parameters, such as the air pressure, blue-light intensities, and the length of the transparent nozzle, the poly(AM/DMAA) hydrogel filaments could be easily extruded and collected.

**Figure 8:**
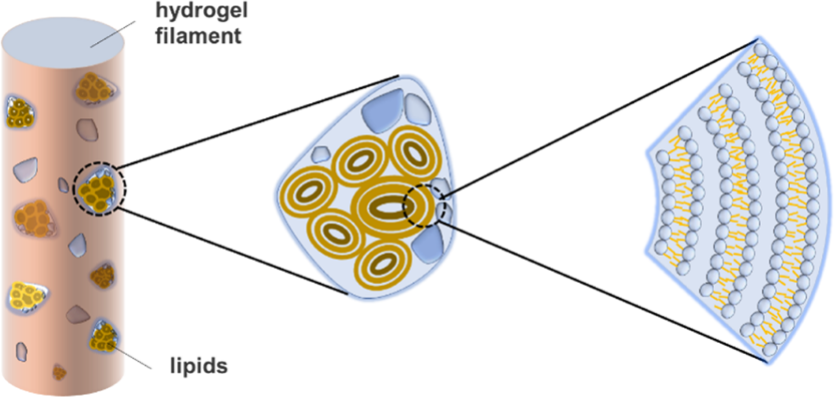
Schematic illustrating the self-lubrication of the lipid-incorporated poly(DMAA/AM) hydrogel filament.

The easy extrusion was mainly attributed to the presence of lecithin, as shown in Figure 8. The relative movement between the hydrogel filament and the internal wall of the nozzle caused the wear of the hydrogel filament. As the surface of the hydrogel, by incorporating lipids as vesicles in microreservoirs, wears away because of friction, additional microreservoirs of lipids are exposed.^27^ This enables boundary layers of lipids to form on the surfaces, leading to friction reduction via the hydration lubrication mechanism at the slip plane between the highly hydrated lipid head groups.

**Figure 9:**
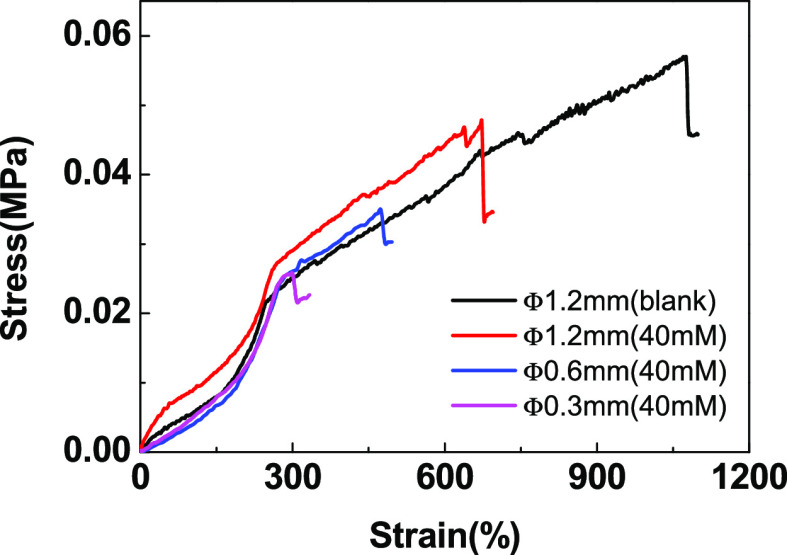
Stress–strain curves of lipid-incorporated poly(DMAA/AM) hydrogel filaments with different diameters.

By controlling the internal diameter of the nozzle, poly(AM/DMAA) hydrogel filaments with different sizes were obtained. The tensile performances of different hydrogel filaments are shown in Figure 9. Clearly, it was found that the fracture stress increased with the increase of the internal diameter of the nozzle, and the stress of the hydrogel filament was improved by introducing lecithin, while its elongation at break was decreased. The stress of the hydrogel filament with 40 mM lecithin and 1.2 mm diameter was nearly 50 kPa, while the elongation at break was over 600%, which indicated a flexible hydrogel filament.

**Figure 10:**
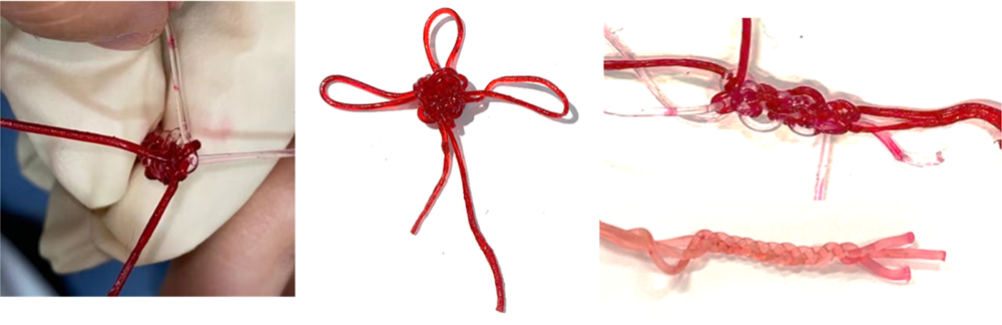
Dyed poly(AM/DMAA) hydrogel filaments and their weavability.

Such a hydrogel filament also presented excellent weavability, as shown in Figure 10. Various complex structures could be formed by weaving multiple hydrogel filaments. Such an ability is believed to be welcomed by scientists in soft matter; further research on applications of such hydrogel filaments is in progress.

## 3 Conclusions

In the present paper, a weavable poly(DMAA/AM) hydrogel filament was realized by a strategy of in situ extrusion and blue-light-photocured technique. Lecithin served as the lubricant, embedded in the poly(AM/DMAA) hydrogel structure. The blue-light photopolymerization efficiency, viscoelasticity, and tensile mechanical properties of poly(AM) were effectively regulated by introducing DMAA into the AM precursor, while lecithin significantly increased the photopolymerization efficiency of the hydrogel precursor and elasticity of the poly(DMAA/AM) hydrogel. The continually reconstructed lipid-based boundary layer at the hydrogel surface in friction and wear ensured the self-lubrication of the poly(DMAA/AM) hydrogel filament. Such a hydrogel filament possesses excellent elasticity and flexibility, which provided a realistic possibility of constructing a complex weaving structure suitable for soft robots, bioengineering scaffolds, and other applications.

## 4 Experimental Section

### 4.1 Materials

**Chart 1:**
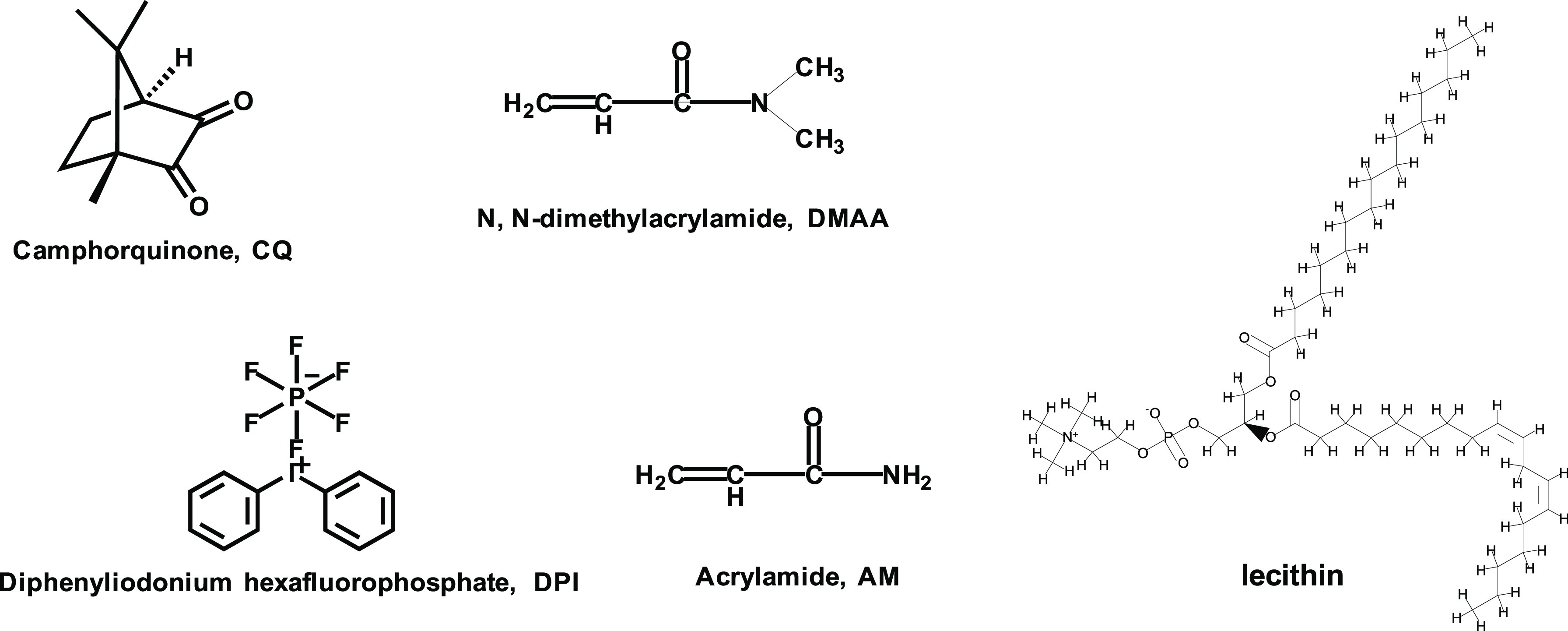
Chemical Structures of CQ, DPI, AM, DMAA, and Lecithin

CQ and the photosensitive synergist diphenyl iodonium hexafluorophosphate (DPI) were purchased from Sigma-Aldrich (St. Louis, MO, USA). AM, DMAA, PEGDA (average *M*_n_ ∼ 1000), and lecithin were acquired from Aladdin Reagent (Shanghai) Co., Ltd. (Shanghai, China). All materials were used as received. Their chemical structures are shown in Chart 1.

### 4.2 Photo-DSC Measurement

Photo-DSC measurements were performed using a differential scanning calorimeter (Q2000, TA Instruments, USA) equipped with a photocalorimetric accessory (PCA OmniCure S2000, EXFO, Canada). Blue-light irradiation from a 200 W mercury arc lamp was delivered by quartz light guides into the DSC cell with a 400–500 nm band-pass filter and a 10% attenuation filter. An uncovered aluminum Tzero pan that contained about 7.0–8.0 mg of the hydrogel sample was used for the test, with another without the sample as a reference. To avoid the inhibition of oxygen in the photopolymerization, ultrahigh-purity nitrogen was used to purge the DSC cell for 5 min and at 50 mL min^–1^ flow rate before the irradiation. Heat flow was recorded in an isothermal mode (25 °C) under various irradiation intensities.

The rate of polymerization and double-bond conversion were calculated using eqs 2 and 3, respectively^28^
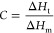
2
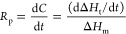
3where *H*_t_ is the enthalpy at time *t* and Δ*H*_m_ is the total enthalpy of polymerization. For acrylic double bond and AM double bond, Δ*H*_m_ = 82 kJ/mol.

### 4.3 Photorheology Measurement

Under the oscillation mode of a rotary rheometer (Anton Paar MCR 52, Austria) also equipped with a photocalorimetric accessory (PCA OmniCure S2000, EXFO, Canada), the evolution of the storage modulus *G*′ and loss modulus *G*″ was acquired during the blue-light polymerization. The oscillatory shear was applied to parallel plates 50 mm in diameter with a 10% strain; this guaranteed the linear viscoelasticity regime during the rheological measurement. The gap between the two plates was 0.10 mm. The input angular frequency during the test was set to 62.832 rad/s at 25 °C. A blue-light irradiation process followed a 2 min oscillatory shear without light. The light intensity was measured by a radiometer (FZ-A, China). The time of gel point (*t*_gel_) is defined as the time when *G*″ is equal to *G*″ after light is triggered.

### 4.4 Tensile Strength Measurement

The tensile strength of poly(DMAA/SA) hydrogel films or filaments was measured according to the ISO 1184-1983 standard and using a universal tester (Instron 3345, USA), with 10 mm/min as the stretch rate (20 mm × 40 mm as the sample size of the hydrogel films).

### 4.5 Extrusion of Poly(DMAA/AM) Hydrogel Filaments

The extrusion system was composed of an air compressor, a brown syringe, a transparent Teflon needle (Φ, 0.3–1.2 mm), and a blue-light photocalorimetric accessory (PCA OmniCure S2000, EXFO, Canada). The poly(DMAA/SA) hydrogel precursor containing lecithin was added into the syringe, and then the air compressor and PCA were activated, and the blue light was ensured to fall on the transparent Teflon needle. The light intensity was measured by a radiometer (FZ-A, China). By adjusting the pneumatic pressure, the blue-light intensity, and the distance between the light source and the syringe, the hydrogel filament could be smoothly extruded through the needle with approx. 0.5 mm/s extrusion speed. The hydrogel filaments were collected in silicone oil.

## Supporting Information Available

Demonstration of hydrogel fiber extrusion molding (MP4)
Demonstration of the loop stretching experiment (MP4)
Polymerization rate and double-bond conversion of the polymerization precursor at different CQ amounts (PDF)
