# Supplementary material for: In Situ Blue-Light-Induced Photocurable and Weavable Hydrogel Filament
Source: ACS Omega. 2021 Dec 16;6(51):35600–6. doi: 10.1021/acsomega.1c05354 (PMC8717588; doi:10.1021/acsomega.1c05354)
Supplement: Supplementary file 3 — ao1c05354_si_003.pdf [file ao1c05354_si_003.pdf]

In-suit Blue Light Induced Photocurable and Weavable Hydrogel Filament

Chenglong Wang, Fan Meng, Luyang Qiao, Yuyan Xie, Xin Liu, Jinhuan Zheng*

Engineering Research Center for Eco-Dyeing and Finishing of Textiles, Ministry of Education, Zhejiang Sci-Tech University, Hangzhou 310018, P. R. China.

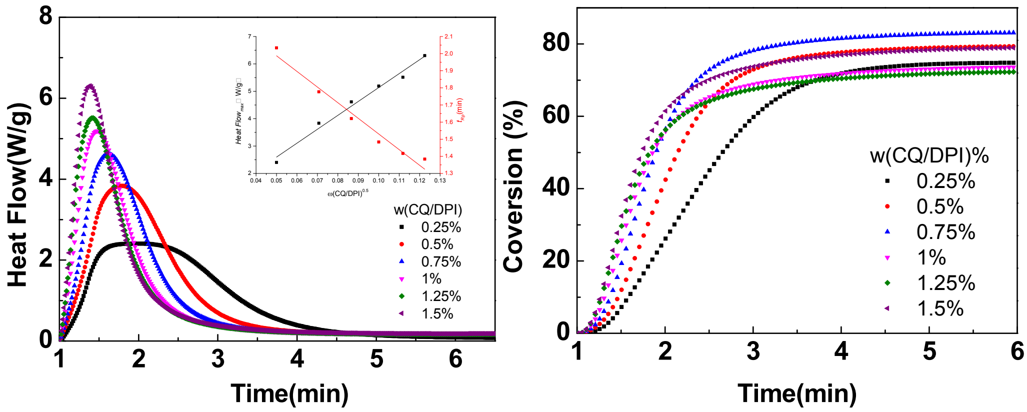

Figure S1. Polymerization rate(A) and double bond conversion(B) of the polymerization precursor at different CQ amounts.
